# Bacteriophage-mediated decolonization of *Klebsiella pneumoniae* in a novel *Galleria mellonella* gut colonization model with* Enterobacteriaceae*

**DOI:** 10.1038/s41598-023-50823-9

**Published:** 2024-01-03

**Authors:** Kamran A. Mirza, Sandor Nietzsche, Oliwia Makarewicz, Mathias W. Pletz, Lara Thieme

**Affiliations:** 1 Jena University Hospital, Institute of Infectious Diseases and Infection Control, Friedrich-Schiller-University Jena, Am Klinikum 1, 07747 Jena, Germany; 2 Jena University Hospital, Leibniz Center for Photonics in Infection Research, Friedrich Schiller University Jena, 07747 Jena, Germany; 3 Center for Electron Microscope, Jena University Hospital, Ziegelmühlenweg 1, 07743 Jena, Germany

**Keywords:** Microbiology, Antimicrobials, Bacteria, Bacteriophages, Pathogens, Infectious diseases

## Abstract

*Galleria mellonella* larvae have emerged as an invertebrate model for investigating bacterial pathogenesis and potential therapies, addressing ethical concerns related to mammalian models. This model has the advantage of having a simple gut microbiome, which is suitable for gut colonization studies. Intestinal colonization by *Enterobacteriaceae* significantly contributes to the spread of antibiotic resistance. This study aimed to establish a novel *Enterobacteriaceae* gut colonization larval model and assess its suitability for evaluating distinct antimicrobial efficacies. Larvae were force-fed sequentially with bacterial doses of *K. pneumoniae* and *E. coli* at 0, 24, and 48 h, with survival monitoring at 24 h intervals. Bacterial counts were assessed after 48 h and 120 h of force-feeding. Successfully colonized larvae were subjected to one-time force feeding of a bacteriophage cocktail (10^7^ PFU/larvae) or MIC-based meropenem and ciprofloxacin. The colonized bacterial load was quantified by CFU count. Three doses of 10^6^ CFU/larvae resulted in stable gut colonization, independent of the *K. pneumoniae* or *E. coli* strain. Compared with the control, force-feeding of the bacteriophage reduced the colonization of the strain Kp 419614 by 5 log_10_ CFU/larvae, while antibiotic treatment led to a 3 log_10_ CFU/larval reduction. This novel *G. mellonella* model provides a valuable alternative for gut colonization studies, facilitating proof-of-concept investigations and potentially reducing or replacing follow-up experiments in vertebrate models.

## Introduction

The intestine is a key reservoir of highly antibiotic-resistant, opportunistic pathogens such as bacteria of the *Enterobacteriaceae* family. *Enterobacteriaceae* have been identified by the World Health Organization (WHO) as a high priority due to their resistance to the last resort antibiotics carbapenems, which can lead to high mortality and a high risk of hospital outbreaks^1^. Intestinal colonization with carbapenemase-producing *Enterobacteriaceae* (CPE), such as *Klebsiella pneumoniae* and *Escherichia coli*, is associated with an increased risk of subsequent nosocomial infections, e.g., pneumonia, urinary tract infection or sepsis. Patients with long-term intensive care unit hospitalization are at high risk of CPE intestinal colonization and breakthrough infections due to weak mucosal immunity, advanced age and prolonged use of antibiotics^2^. Thus, selective mitigation of the gut from CPE might prevent downstream lethal infections. However, many CPEs are resistant to other antibiotic classes suitable for gut treatment. Furthermore, antibiotics lack host specificity, resulting in dysbiosis of the gut microbiome and an increased risk of difficult-to-treat infections caused by *Clostridium difficile*^3^. An alternative approach is therefore the therapeutic application of bacteriophages.

Bacteriophages are bacterial viruses that effectively kill bacteria during their lytic cycle. They can have very high specificities not only for one bacterial species but also for a certain subspecies or even strain and thus represent a highly interesting type of treatment to specifically address a certain pathogen^4^. Although bacteriophages were discovered more than a century ago, the last decade has greatly explored their therapeutic potential. Bacteriophages possess several benefits over antibiotics, such as host specificity, which means that they do not affect the microbiota or mammalian cells^4^. Another benefit of bacteriophage therapies is their self-limiting effects, which include the cessation of bacteriophage activity when the bacterial host is eliminated^4^. Bacteriophages were successfully applied intranasally and topically in mice against *K. pneumoniae*-associated lung and wound infections, revealing decreased bacterial loads and mortality rates^5,6^. A recent study also identified two lytic phages capable of intestinal decolonization of *K. pneumoniae* in a mouse model^7^.

To overcome strong ethical concerns due to the high burden of disease in mammalian infection models, researchers have developed alternative invertebrate models, such as the *G. mellonella* larval infection model. Following the 3Rs framework in animal research (Replacement, Reduction, Refinement)^8^, larval models allow for high-throughput and proof-of-concept screens, leading to a reduction in the number of animals in subsequent mammalian experiments. Larval research models in general are associated with low costs and are easy to manipulate. Thus, in contrast to mammalian experiments, specialized training, complex animal husbandry and ethical approval are not needed. The advantages of *G. mellonella* larvae compared to those of other invertebrate models include a comparatively similar innate immune response to mammals, susceptibility to human pathogens, and survival at a temperature range of 37 to 42 °C^9^. *G. mellonella* larvae can be easily inoculated orally or via their abdominal proleg structure. The gradual melanization as an immune response to a pathogen allows easy monitoring of the course of infection in virulence studies and efficacy studies of antimicrobial agents.

Numerous studies have utilized the *G. mellonella* model to study enteric pathogens such as *Yersinia pseudotuberculosis*, *Campylobacter jejuni*, *Yersinia enterocolitica*, and *Shigella* spp.^10–13^. Most of these studies induced systemic infection by injecting bacteria into the larval hemolymph rather than orally. Oral injection has been described previously^14^, but the focus has been on characterizing induced innate immune responses instead of stable gut colonization. The gut of native larvae has further been characterized by microbiome studies^15^. Various studies have evaluated the efficacy of bacteriophages against systematic infection by *K. pneumoniae*^16–19^ but thus far not against gut-colonizing bacteria. To the best of our knowledge, insect studies on the decolonization of CPE from the gut are lacking due to the absence of a well-established *G. mellonella* gut colonization model. Therefore, this study aimed to establish a stable gut colonization model in *G. mellonella* larvae using *K. pneumoniae* and *E. coli*, both common CPEs, and to assess the antimicrobial effects of these bacteria on reducing CPE loads in the gut.

## Animals, materials and methods

### Selection and culture of bacterial strains

Two clinical isolates and one laboratory standard for each species were used for the studies (Table 1). The clinical isolates were previously collected in studies approved by the ethical committee of Jena University Hospital (3852/07-13 and 3694-02/13). The study is reported in accordance with the applicable ARRIVE guidelines^20^. The minimum inhibitory concentrations (MICs) of the antibiotics were routinely assessed according to the European Committee of Antimicrobial Susceptibility Testing (EUCAST) via VITEK2 (bioMérieux, Marcy-l’Étoile, France) and interpreted according to the clinical EUCAST breakpoints in 2022. In general, all methods were performed in accordance with the relevant guidelines and regulations. Bacteria were cultured in Müller Hinton (MH) broth (Oxoid Deutschland GmbH, Wiesel, Germany) at 37 °C in a shaking incubator for 2 h to reach the early exponential phase.

**Table 1:** Bacterial strains used for colonization studies and their measured MIC values.

Strain	*K. pneumoniae* (mg/L)			*E. coli* (mg/L)		
	ATCC 700603	Kp 14520	Kp 419614	ATCC 35218	Ec 208873	Ec 280624
Specimen	LS	Urine	Stool	LS	Urine	Stool
Amoxicillin	8	32 (R)	–	8	32 (R)	32 (R)
Fosfomycin	–	64 (R)	–	2	64 (R)	64 (R)
Piperacilline/tazobactam	16	16 (I)	128 (R)	1	4 (I)	128 (R)
Ceftazidime	32	32 (R)	64 (R)	–	4 (R)	64 (R)
Meropenem	0.5	0.06 (S)	32 (R)	< 0.015	0.25 (S)	2 (R)
Ciprofloxacin	4	32 (R)	32 (R)	> 1	4 (R)	4 (R)
Gentamicin	8	-	8 (R)	4	1 (S)	1 (R)

*LS* laboratory standard, sensitive (S), resistant (R), intermediate (I).

### Systemic infection of *G. mellonella* larvae via the hemolymph

*G. mellonella* wax moth larvae were obtained from Bruno Mariani-FLOTEX (Augsburg, Germany). Larvae were in the 5th instar stage and stored in a refrigerator at 15 °C for a maximum of 2 weeks. Throughout the experiments, only agile larvae with an average size of 3.3 cm ± 0.12 cm (mean and standard deviation (SD)) were used. Larvae were divided into five groups (n = 5 × 2 = 10 per dose), with each group receiving log_10_-fold increasing concentrations of *K. pneumoniae* 14520, i.e., 10^2^ CFU/larvae to 10^6^ CFU/larvae. From the early exponential phase, the bacterial suspension was centrifuged, washed twice with PBS, and adjusted to 10^8^ CFU/mL in PBS, corresponding to 10^6^ CFU/larvae. Bacterial doses were injected through the middle proleg into the larvae using a 10 µL ALS syringe, and the larvae were incubated at 37 °C. The survival of the larvae was observed every 24 h, and Kaplan‒Meier curves were generated.

### Gut colonization of *G. mellonella* larvae

**Figure 1:**
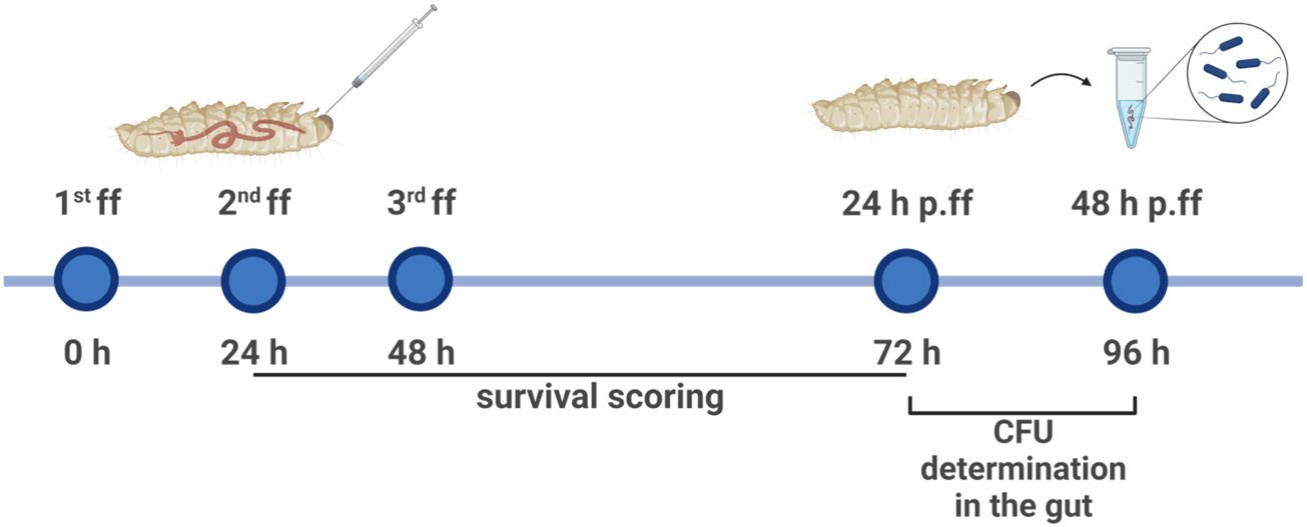
Timeline of the methodology adopted for the establishment of gut colonization. *ff* force feed, *p.ff* post force feed.

For proof-of-concept, a bacterial suspension of Kp14520 was taken from the early exponential phase and adjusted to 10^8^ CFU/mL in MH broth. Larvae in groups of n = 20 × 3 per bacterial dose were gavaged with logarithmically increasing doses of bacteria from 10^2^ to 10^6^ CFU/larvae. Force-feeding (ff) was performed at 0 h, 24 h, and 48 h using a 10 µl ALS syringe (Agilent Technologies, Santa Clara, USA) under a Stemi 2000-C microscope (Carl Zeiss, Oberkochen; Germany) (Fig. S1) followed by incubation at 37 °C (Fig. 1). Survival of the larvae was monitored 24 h, 48 h and 72 h post ff, and Kaplan–Meier curves were generated. After 24 h and 48 h of gavage, i.e., 72 h and 96 h after the first dose of bacteria, the larvae were anesthetized on ice for 30 min, and the gut was surgically removed and transferred to 300 µL of 1× phosphate buffer saline (PBS) (Carl Roth GmbH, Karlsruhe, Germany). The gut was crushed thoroughly using a dissecting needle lancet, vortexed for 1 min, serially diluted, and plated. For CFU counting, MacConkey agar (Becton Dickinson, New Jersey, USA) supplemented with 1 mg/L ciprofloxacin (PanReac AppliChem, Darmstadt, Germany) was used as a selective media to inhibit the growth of the *Enterococcus*-dominated microbiome of the larvae^15^. To evaluate the interstrain variability in intestinal colonization, two additional *Klebsiella* strains, i.e., 10^5^ and 10^6^ CFU/larvae of Kp 419614 and ATCC 700603, were gavaged on the larvae following the same protocol. The larvae were also colonized with *E. coli* ATCC 35218, Ec 280624 and Ec 208873 to determine the variation among different *Enterobacteriaceae* species. To confirm the prolonged colonization stability of *K. pneumoniae* and *E. coli* strains in the larval gut, a group of larvae (n = 5 × 2 = 10 per bacterial dose) was gavaged three times with 10^6^ CFU/larvae and incubated for 120 h post ff, i.e., 168 h after the first dose of bacteria. After 120 h of incubation, the same protocol for CFU counting was used as described above.

### Decolonization of *K. pneumoniae* by antibiotics and phages

The larval gut colonization model was used to investigate the efficacy of different antimicrobial agents against *K. pneumoniae* isolates. Ciprofloxacin (PanReac AppliChem, Darmstadt, Germany) and meropenem (TCI Germany GmbH, Eschborn, Germany) were freshly prepared as 10 mg/mL stock solutions in sterile distilled water before use. One day after successful colonization (i.e., 24 h p.ff. (Fig. 1)), the larvae were gavaged once with antimicrobial agents, and the concentrations were decreased in MH media corresponding to the isolate-specific minimum inhibitory concentrations (MICs) (Table 1). After 24 h of incubation, the larvae were anesthetized, and a single-cell suspension of the gut was plated as described above.

A combination of bacteriophages was also used to evaluate the antimicrobial effectiveness of the model. The bacteriophage methodology was conducted in accordance with previously published guidelines^21^. The bacteriophages UZG4 and UZG13 (Table 2) and their propagation host *K. pneumoniae* 1711-O4741 were obtained from Ghent University Hospital, Belgium. For propagation and harvesting of the phages, a culture of *K. pneumoniae* 1711-O4741 was grown overnight at 37 °C and 160 rotations per minute (RPM). This bacterial suspension was mixed 3:1 with the phage lysate and incubated at 37 °C and 160 RPM for 30 min. Next, top agar (0.5% agar in Luria–Bertani (LB) broth (Oxoid Germany GmbH)) was added to the mixture, vortexed and uniformly poured onto LB agar (Oxoid Germany GmbH) plates, followed by overnight incubation. Subsequently, LB broth was pipetted onto the agar plate and incubated at 37 °C and 80 rpm in an orbital shaker for 2 h. After the incubation, the lysate and the top agar were harvested and centrifuged at 10,000 relative centrifugal force (RCF) for 5 min. The supernatant was collected and filtered via a 0.2 µm syringe filter (Buch & Holm A/S, Herlev, Denmark). The phage titer was evaluated using double agar overlay assays^22^. The phage stocks were adjusted in LB broth to a titer of 10^10^ plaque-forming units (PFU)/mL and stored at 4 °C. For decolonization experiments, a bacteriophage cocktail comprising both phages with a 10^7^ PFU/larvae titer was gavaged one time to successfully colonized larvae 24 h post force feeding (Fig. 1). The susceptibility of the isolates, such as those with high susceptibility to ATCC 700603 and Kp 419614 and less susceptibility to the Kp 14520 isolate, differed (Fig. S3). After 24 h of incubation, the remaining bacterial load in the gut was determined as described above.

**Table 2:** Bacteriophages used in the study.

Phage name	Bacterial host for propagation	Phage morphology	Accession number	Classification
UZG4	*K. pneumoniae* 1711-O4741	Myoviridae	KT239446.1 (https://www.ncbi.nlm.nih.gov/nucleotide/KT239446.1?report=genbank&log$=nucltop&blast_rank=1&RID=0B99WUHD01R)	Jiaodavirus
UZG13	*K. pneumoniae* 1711-O4741	Myoviridae	GU295964.1 (https://www.ncbi.nlm.nih.gov/nucleotide/GU295964.1?report=genbank&log$=nucltop&blast_rank=5&RID=0B9JB0KF01R)	Slopekvirus

### Transmission electron microscopy

The bacteriophages on the bacterial cells were visualized using transmission electron microscopy (TEM) (Fig. S3). A 0.5 McFarland bacterial suspension of ATCC 700603 was mixed with 10^7^ PFU/mL bacteriophage cocktail of UZG4 and UZG13. The suspension was incubated at 37 °C at 160 rpm with shaking for 4 h. After the incubation, the suspension was centrifuged at 10,000 RCF for 1 h. The pellet was fixed with freshly prepared 2.5% (v/v) glutaraldehyde using 2.5% glutaraldehyde (SERVA Electrophoresis GmbH, Heidelberg, Germany) mixed with 0.1 M sodium cacodylate buffer (pH 7.4, SERVA Electrophoresis GmbH, Heidelberg, Germany) for 1 h at room temperature. After washing 3 times for 15 min each with 0.1 M sodium cacodylate buffer (pH 7.4), the pellets were post-fixed with 2% (w/v) osmium tetroxide for 1 h at room temperature. During the following dehydration in an ascending ethanol series, post staining with 1% w/v uranyl acetate for 1 h was performed. Afterward, the pellets were embedded in epoxy resin (Araldite) and sectioned using a Leica Ultracut S (Leica, Wetzlar, Germany). Finally, ultrathin sections were mounted on filmed Cu grids, post stained with lead citrate, and studied under a transmission electron microscope (EM 900, Zeiss, Oberkochen, Germany) at 80 kV and a magnification of 20,000×. For image recording, a 2K slow-scan CCD camera (TRS, Moorenweis, Germany) was used.

### Statistical analysis

All the data were processed and analyzed using GraphPad Prism 9 (GraphPad Software, Inc., San Diego, USA). The Kaplan‒Meier curves were compared using the log-rank (Mantel–Cox) test. The gut colonization and decolonization CFU counts were analyzed using the Kruskal‒Wallis test. Two-sided confidence intervals of 5–95% were assumed. *P* values < 0.05 were considered to indicate statistical significance.

## Results

### Survival and colonization stability of larvae

**Figure 3:**
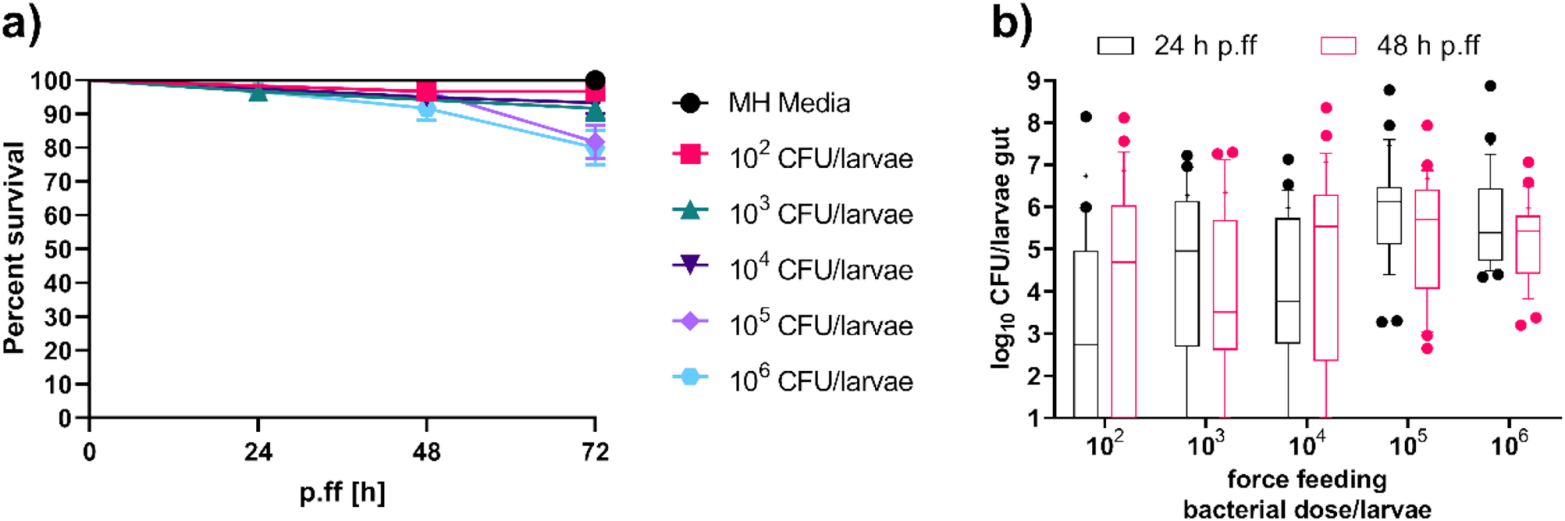
Survival curves of the force-fed larvae (**a**) and CFU counts of the bacteria that had colonized after 24 h and 48 h of last force-feeding (**b**) with increasing doses of Kp14520. (**a**) Larvae were force-fed at 0 h, 24 h, and 48 h, and the bacterial doses are indicated in the legend. The survival of the larvae was noted at 24 h, 48 h, and 72 h post force feeding. Each experiment was repeated thrice with 20 larvae per group (n = 60), and the data were analyzed with the log-rank (Mantel–Cox) test (results depicted in Table S1). (**b**) Box and whisker plot of the gut colonization of the different larval groups after 24 h and 48 h of last force-feeding. The Gram-negative bacteria were selected on MacConkey agar. The experiment was performed thrice, with six larvae per group on the first attempt and ten larvae per group on the second and third attempts (n = 26). The data were analyzed with the Kruskal‒Wallis test (the results are shown in Table S1). The boxes represent the 90th-10th percentiles, the line represents the median, + represents the mean and dots represent values outside the percentile.

Larvae were fed increasing concentrations of bacteria to determine the optimal dose for colonization in the larval gut without causing high mortality. The Kp14520 isolate was force-fed thrice to the larvae at different doses, and ≥ 80% of the larvae survived colonization even in the highest dose group. At 72 h, the survival rate was 97% with a force-feeding dose of 10^2^ CFU/larvae and 80% with a bacterial dose of 10^6^ CFU/larvae (Fig. 3a). Compared with those of the control (MH media) and 10^2^ CFU/larvae, the bacterial concentrations of 10^5^ CFU/larvae and 10^6^ CFU/larvae had significantly different survival rates (Table S1).

The colonization stability was assessed by determining the CFU count in the gut of live larvae across dosage Groups 24 and 48 h after the last feeding. All the larval groups colonized, regardless of the bacterial dose, but with varying levels of variability (Fig. 3b). The bacterial dosages of 10^5^ and 10^6^ CFU/larvae displayed the least variability in CFU count, indicating more stable colonization than at lower dosages. The median CFU counts were approximately similar to the amount of bacteria fed at each dosage. The bacterial concentrations of 10^5^ CFU/larvae and 10^6^ CFU/larvae were significantly different in terms of CFU count compared to 10^2^ CFU/larvae (Table S1). Furthermore, compared with those of 10^3^ CFU/larvae, 10^5^ CFU/larvae exhibited significant differences (Table S1). As a result, subsequent colonization experiments focused on doses of 10^5^ and 10^6^ CFU per larvae due to the low variability and notable differences in CFU count compared to those of lower doses.

**Figure 4:**
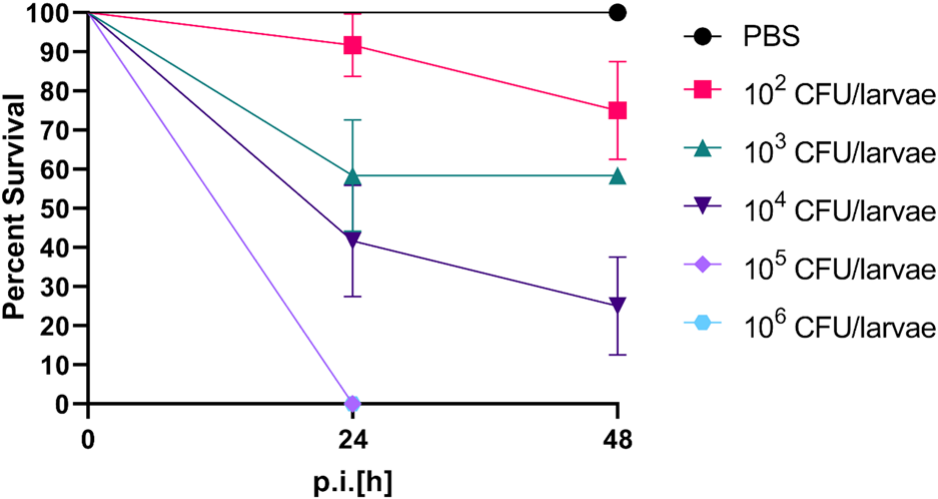
Kaplan–Meier curves of the larvae groups injected with bacterial doses in the hemolymph through the proleg. The 5 groups of larvae were injected with 10^2^, 10^3^, 10^4^, 10^5^ or 10^6^ CFU/larvae. The experiment was repeated twice with 6 larvae per group (n = 12), and the results were analyzed with the log-rank (Mantel–Cox) test (the results are shown in Table S1). The error bars represent the standard error (SE).

Following successful colonization, the larvae were injected with different doses of Kp14520 into the hemolymph to investigate whether previous colonization occurred due to the non-virulent behavior of the isolate toward the larvae. The results showed that 10^6^ CFU/larvae and 10^5^ CFU/larvae were sufficient to kill all the larvae within 24 h of inoculation (Fig. 4). Survival rates of 58% and 25% were observed for infection dosages of 10^3^ and 10^4^ CFU/larvae, respectively. The minimum infection dose of 10^2^ CFU/larvae led to a survival rate of 80%, similar to three rounds of force-feeding with 10^6^ CFU/larvae (Fig. 3a). Significant differences in survival were found between the 10^3^, 10^4^, 10^5^, and 10^6^ CFU/larvae-injected groups and the PBS-injected group (Table S1).

**Figure 5:**
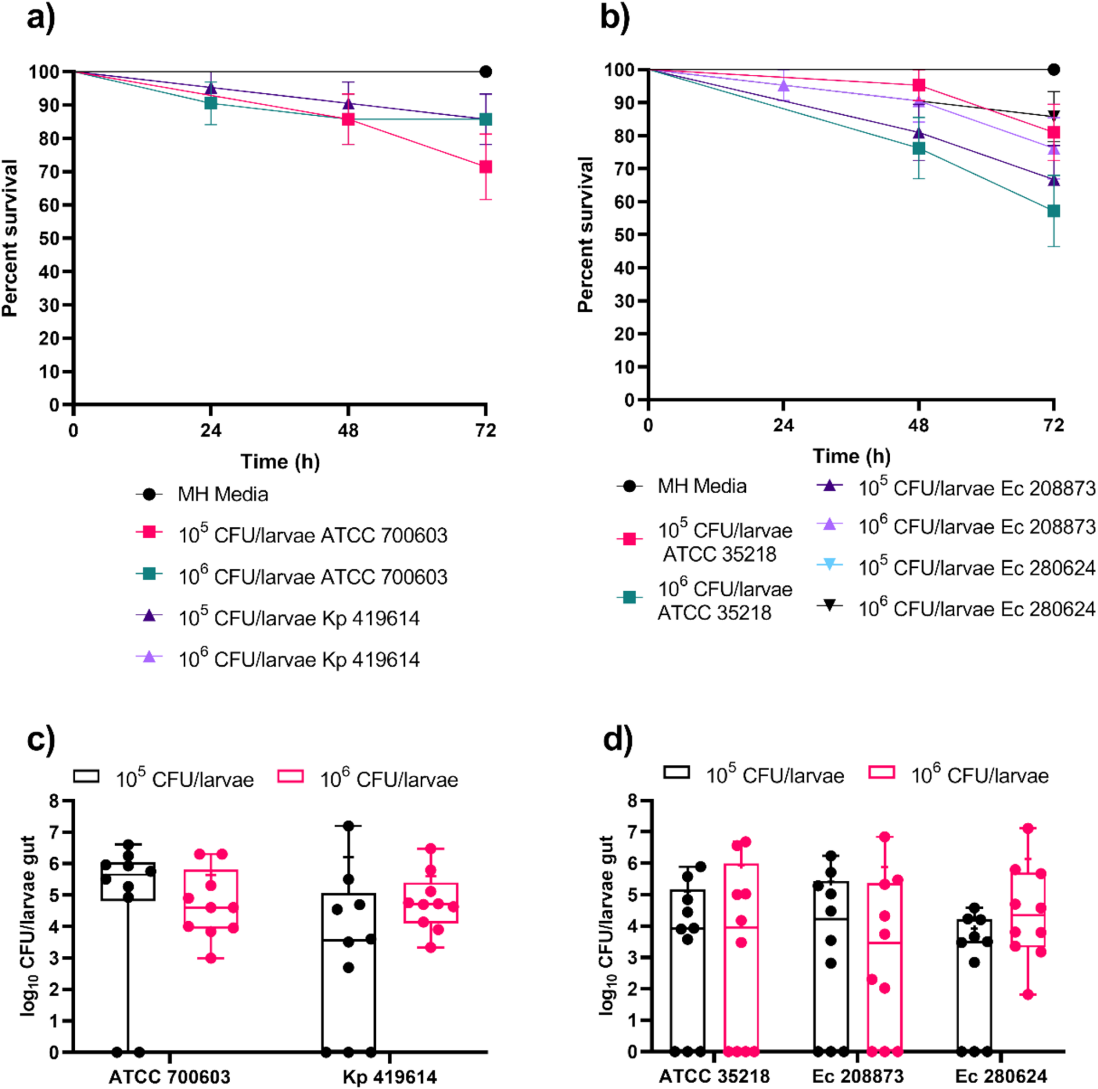
Survival curve of colonized larvae (**a**) and CFU counts 48 h after the last force feeding of the larvae gut colonized with *K. pneumoniae* ATCC 700603 and Kp 419614 (**c**). Survival curve of colonized larvae (**b**) and CFU count after 48 h of last force feeding of the larval gut colonized with *E. coli* ATCC 35218, isolates Ec 208873 and Ec 280624 (**d**). Larvae were colonized with 10^5^ and 10^6^ CFU/larvae bacterial doses of *K. pneumoniae* and *E. coli* strains (**a,b**). Three-force feedings were performed, and survival was observed every 24 h. The experiment was repeated three times (6 × 2 + 9 × 1 = 21), and the data were analyzed with the log-rank (Mantel–Cox) test (the results are presented in Table S1). The error bars represent the standard error (SE). Larvae were divided into groups based on the bacterial dose applied to the larvae via force feed (**c,d**). After 48 h of gavage, the larval guts were isolated. MacConkey agar was used for selection of Gram-negative bacteria. The experiment was performed twice, with five larvae per group (n = 10). The boxes represent 75th percentiles, the whiskers represent the minimum to maximum, the line represents the median, + represents the mean and the dots represent all the data points.

The effect of isolate and bacterial species variation on the stability of colonization was evaluated by colonizing larvae with either 10^5^ or 10^6^ CFU/larvae of Kp 419614, ATCC 700603, ATCC 35218, Ec 208873 or Ec 280624. The survival rate of larvae harboring *K. pneumoniae* strains was 80%, except for the larvae in the group fed ATCC 700603, which received a bacterial dose of 10^5^ CFU/larvae and had a survival rate of 70% (Fig. 5a). A significant difference in survival was observed between ATCC 700603 10^5^ CFU/larvae and gavaged larvae in MH media (Table S1). Compared to those of the *K. pneumoniae* strains, the larval groups colonized with the *E. coli* strains exhibited greater mortality, with 50% and 60% survival recorded for ATCC 35218 and 280624, respectively (Fig. 5b). Compared with those on the MH media, the survival of larvae force-fed with 10^5^ CFU/larvae or 10^6^ CFU/larvae of the *E. coli* strains significantly differed (Table S1). CFU counts were determined 48 h after gavage for all larval groups, and colonization was confirmed. The median colonization of the larvae with *K. pneumoniae* strains was 5 log_10_ CFU/larval with a bacterial dose of 10^6^ CFU/larvae (Fig. 5c). Larvae colonized with *E. coli* strains had lower median CFU counts (Fig. 5d). When larvae were fed 10^6^ CFU/larvae of *E. coli* strains, the median CFU count was 4 log_10_ CFU/larvae. However, the mean CFU count of larvae that were gavaged with 10^6^ CFU/larvae of the *E. coli* strains was the same as that of the force-fed larvae. Notably, some of the larvae were colonized with neither *K. pneumoniae* nor *E. coli*, regardless of the bacterial strain (data points on the x-axis line). The use of a bacterial dose of 10^6^ CFU/larvae had less variation than the use of 10^5^ CFU/larvae, so subsequent experiments were performed with a 10^6^ CFU/larvae dosage.

**Figure 6:**
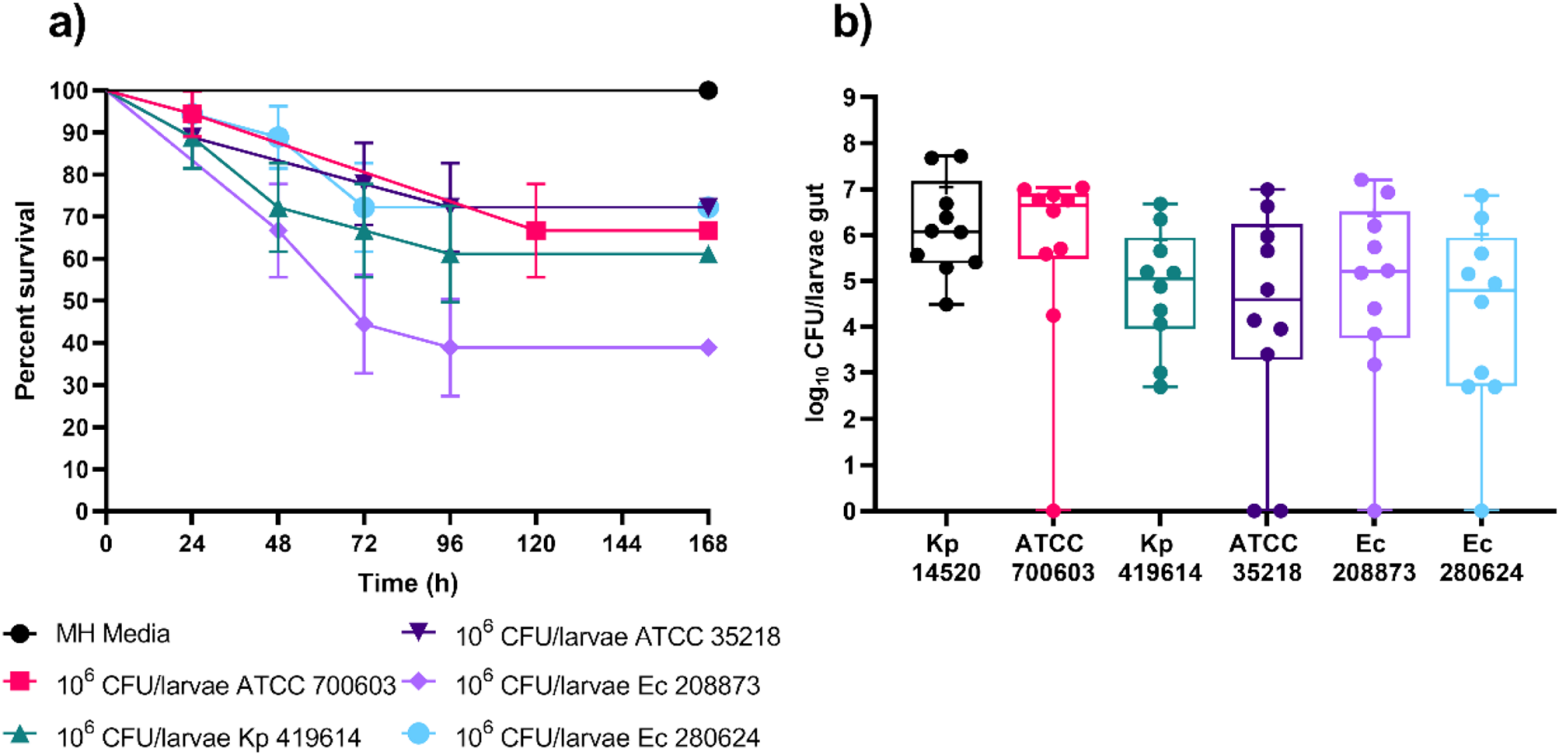
Survival curve of larvae colonized (**a**) and CFU count after 120 h of last force feeding (**b**) of the larval gut colonized with *K. pneumoniae* and *E. coli* strains. Larvae were colonized with 10^6^ CFU/larvae bacterial dose. Larvae were force-fed three times and monitored for survival every 24 h. The experiment was repeated three times (6 × 3 = 18), and the data were analyzed with the log-rank (Mantel–Cox) test (results are depicted in Table S1). The error bars represent the standard error (SE). Larvae were divided into different groups based on the force-fed to the larvae (**b**). After 120 h of last force-feeding, the larval gut was isolated and plated for CFU counting on MacConkey agar. The experiment was performed twice, with five larvae per group (n = 10). The boxes represent the 75th percentile, the whiskers represent the minimum and maximum values, the line represents the median, the + represents the mean and the dots represent all the data points.

The long-term stability of colonization and survival of the larvae were assessed by gavage with a threefold bacterial dose of 10^6^ CFU/larvae of *K. pneumoniae* and *E. coli* strains each and incubation for 120 h after the last force-feeding cycle. Survival rates decreased by 10–20% compared to those at 48 h (Fig. 5). The different strains had varying impacts on survival: ATCC 700603, Kp 419614, ATCC 35218, and Ec 280624 resulted in 67%, 61%, 72%, and 72% survival, respectively (Fig. 6a). Notably, larvae colonized with Ec 208873 exhibited the lowest survival at 39% after 120 h. The survival of the larvae colonized with *K. pneumoniae* and *E. coli* strains was significantly different from the survival of the larvae in force-fed MH media (Table S1). Despite the lower survival rates, the surviving larvae maintained colonization, with *K. pneumoniae* occurring at 6–7 log_10_ CFU and *E. coli* occurring at approximately 5 log_10_ (Fig. 6b). The colonization rate remained stable even after incubating for 120 h. This finding suggested stable colonization after 24 h, 48 h and 120 h, with CFU counts remaining steady at approximately 6 log_10_ throughout. The *K. pneumoniae* strains showed less variation than the *E. coli* strains, so further decolonization experiments were only performed with *K. pneumoniae* strains.

### Larval gut colonization model for antimicrobial efficacy assessment

The gut colonization model was assessed to evaluate the efficacy of different antimicrobial agents, such as meropenem, ciprofloxacin and bacteriophage cocktail, against the colonized *K. pneumoniae* isolates.

**Figure 7:**
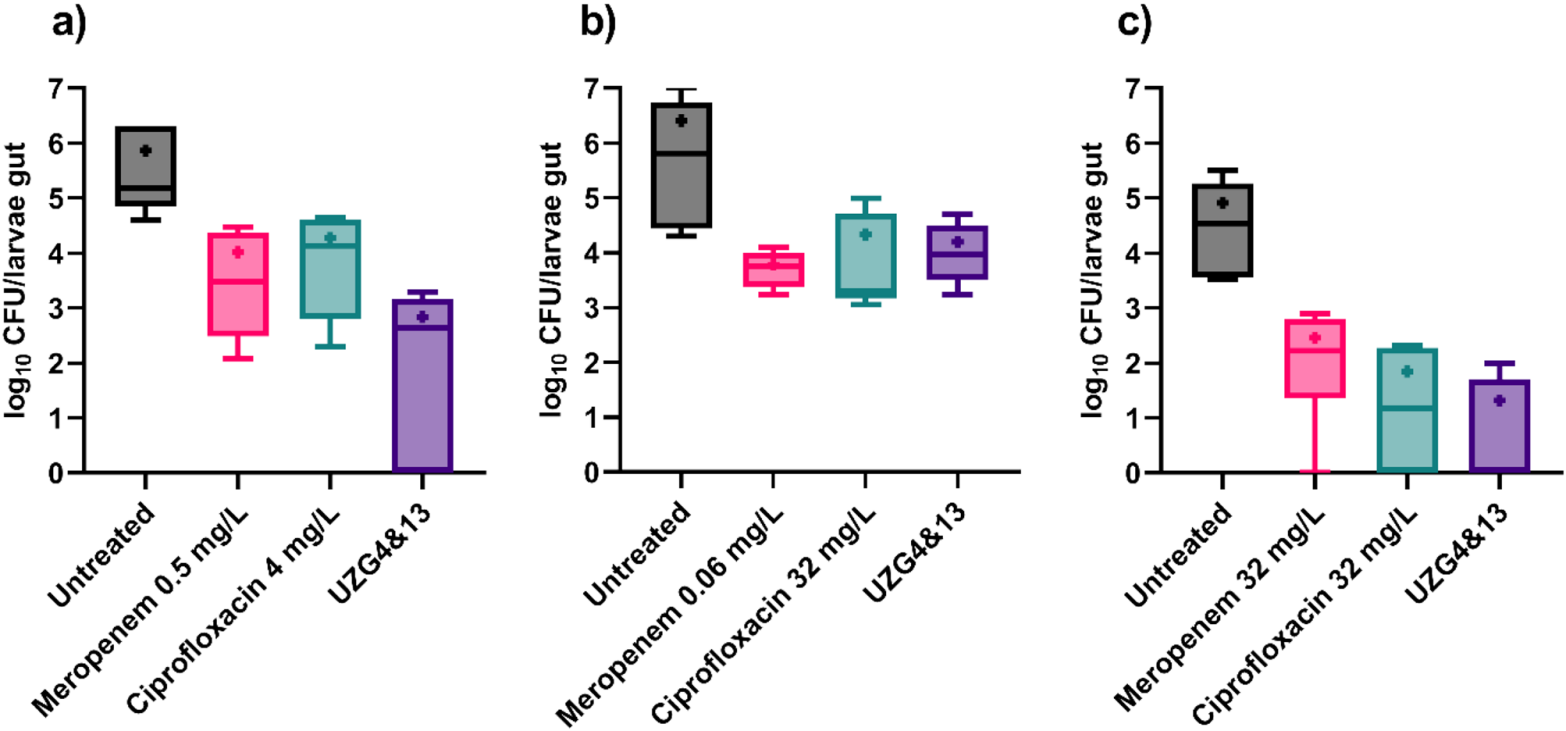
Assessment of antimicrobial efficacy in reducing larval colonization by Kp strains such as ATCC 700603 (**a**), Kp 14520 (**b**) and Kp 419614 (**c**). After colonization, the larvae were one-time force-fed with 10^7^ PFU/larvae bacteriophage cocktail and MIC-based antibiotics and incubated for 24 h. CFU counts were evaluated after incubation. The experiment was repeated twice with 3 larvae per group (n = 6). The statistical significance of the differences was determined using the Kruskal‒Wallis test (the results are shown in Table S1). The outliers were removed using the ROUT test (Q = 1%). The boxes represent the 90th to 10th percentiles, the line represents the median, and + represents the mean.

The findings demonstrated that the larvae colonized with ATCC 700603 showed a significant reduction of 3 log_10_ CFU/larvae when the larvae were treated with the bacteriophage cocktail (Fig. 7a). Conversely, in the group of larvae colonized with Kp14520, significant reductions were observed when the larvae were treated with both meropenem and ciprofloxacin (Fig. 7b). Although the bacteriophage also decreased the CFU count in this group, the difference was not statistically significant. For the larvae colonized with Kp 419614, ciprofloxacin and a bacteriophage cocktail led to significant reductions of approximately 3 log_10_ and 5 log_10_ CFU/larvae, respectively. All the treatments were administered once orally, and no significant difference was observed between the antimicrobial groups. Mock treatment with the antimicrobial agents ensured 100% survival of the larvae.

## Discussion and conclusion

This study investigated for the first time the use of *G. mellonella* larvae as a model for gut colonization by *K. pneumoniae* and *E. coli*. In addition, one-time distinct antimicrobial agents were administered through force-feeding to assess the model for the evaluation of antimicrobial efficacy by reducing *K. pneumoniae* strain colonization in the larval gut. The results showed that *K. pneumoniae* and *E. coli* were able to colonize the larval gut for 120 h after force feeding in the majority of the larval population and with different bacterial strains. Furthermore, the use of antimicrobial agents revealed the potential of this model for evaluating the effect of drugs on selectively colonized bacteria.

Larvae colonized with different *Enterobacteriaceae* exhibit varying mortality rates over time. For instance, the colonization of *E. coli* resulted in higher mortality than that of *K. pneumoniae*. These differences in pathogenicity among various bacteria can be attributed to multiple factors^13^. One key factor is variation in the host immune system, which can lead to varying levels of susceptibility to a particular pathogen. Additionally, the genetic makeup of the bacteria may also play a role in their pathogenicity, as certain bacteria may have evolved to be more harmful to specific hosts. In the case of *G. mellonella*, the microbiome is predominantly composed of *Enterococcus* species^15^, with the absence of *K. pneumoniae* and *E. coli*. Consequently, the larvae demonstrated greater tolerance to Gram-positive bacteria than to Gram-negative bacteria.

The observed CFU counts exhibited notable variation, with certain larvae displaying no measurable CFU. This discrepancy may be attributed to several factors, including the inherent variability among individual larvae, such as their robust immune systems, competitive exclusion by the larvae’s microbiome, or unavailability of a standardized research line of *G. mellonella*. Furthermore, the larvae were kept in a starved state throughout the colonization process during the experiments. This specific condition likely contributed to the lack of a discernible increase in average colonization over time. Nonetheless, successful and stable colonization of the larvae's gut was achieved with all the bacterial strains.

The colonization of MDR Enterobacteriaceae bacteria in the gut, especially during prolonged hospital stays, significantly increases the risk of nosocomial infections. Notably, ESBL-producing bacteria such as *E. coli* and *K. pneumoniae* are concerning due to limited antibiotic treatment options. Upon the emergence of resistance to both ciprofloxacin and meropenem, the spectrum of viable antibiotic interventions has markedly decreased. Furthermore, antibiotics are known to have devastating effects on the healthy microbiome. Antibiotic treatment can reduce microbial diversity^23^, eradicate beneficial microbes^24^ and promote antibiotic resistance^25^. The surge in antibiotic resistance has revived interest in bacteriophage therapy. Recent decolonization studies have used mouse models to investigate the efficacy of bacteriophage therapy for decolonizing intestinal *K. pneumoniae*. Upon colonization, the mice were subsequently treated with different concentrations of phages. Compared with both antibiotic treatment and no treatment, phage treatment resulted in significantly decreased levels of *K. pneumoniae* in the gut microbiota^26^. In our study, the one-time phage cocktail was able to reduce the colonization of *K. pneumoniae* strains within the gut in comparison to antibiotics and the untreated control while simultaneously not killing the larvae. Meropenem and ciprofloxacin are both commonly employed antibiotics for nosocomial infections caused by gut-colonized multidrug-resistant bacteria. Notably, all of the bacterial isolates used in this study were resistant to ciprofloxacin according to the EUCAST breakpoint guide 2022. The antibiotic concentrations used in this study were MIC-based, which is one of the reasons that the administration of antibiotics also significantly reduced the number of colonized bacteria. The preliminary outcomes of the bacteriophage treatment revealed promising results in gut decontamination. Despite the advantageous specificity of bacteriophages over antibiotics, assessing the overall treatment efficacy is challenging due to the limitations of the study. The lack of evaluation of the antimicrobial effects on the microbiome and the necessity of investigating resistance mechanisms require further exploration to comprehensively compare treatment outcomes.

*G. mellonella* is an effective alternative model for gut colonization studies. We introduced a novel approach in which three force feedings were used to colonize *G. mellonella* larvae. This protocol capitalizes on the simplicity, speed, and replicability of the larval model. Although not a complete replacement for mammalian models, the larval model offers advantages, reducing unnecessary mouse use and serving as an effective research alternative. Notably, bacteriophage interventions exhibit the potential to reduce the abundance of colonized bacteria in the larval gut. However, the primary focus of this study was to assess the suitability of *G. mellonella* as a gut colonization model. To attain a more comprehensive antimicrobial impact on colonized bacteria, additional research is needed to validate this model to evaluate antimicrobial efficacy.

### Supplementary Information

Supplementary Information.

## Data Availability

All the data generated in the study are included in the manuscript and can be passed on to interested parties upon request. The data supporting the findings of this study are available upon request. For access, please contact the corresponding author, Kamran Mirza.

## References

[1] Tacconelli E (2018). Discovery, research, and development of new antibiotics: The WHO priority list of antibiotic-resistant bacteria and tuberculosis. Lancet Infect. Dis..

[2] Halpin AL (2016). Intestinal microbiome disruption in patients in a long-term acute care hospital: A case for development of microbiome disruption indices to improve infection prevention. Am. J. Infect. Control.

[3] Ghose C (2013). *Clostridium difficile* infection in the twenty-first century. Emerg. Microbes Infect..

[4] Gordillo Altamirano FL (2019). Phage therapy in the postantibiotic era. Clin. Microbiol. Rev..

[5] Cao F (2015). Evaluation of the efficacy of a bacteriophage in the treatment of pneumonia induced by multidrug resistance *Klebsiella pneumoniae* in mice. Biomed. Res. Int..

[6] Kumari S (2011). Bacteriophage versus antimicrobial agents for the treatment of murine burn wound infection caused by *Klebsiella pneumoniae* B5055. J. Med. Microbiol..

[7] Fang Q (2022). Characterization of phage resistance and phages capable of intestinal decolonization of carbapenem-resistant *Klebsiella pneumoniae* in mice. Commun. Biol..

[8] Tannenbaum J (2015). Russell and Burch’s 3Rs then and now: The need for clarity in definition and purpose. J. Am. Assoc. Lab. Anim. Sci..

[9] Ménard G (2021). *Galleria mellonella* as a suitable model of bacterial infection: Past, present and future. Front. Cell. Infect. Microbiol..

[10] Champion OL (2009). *Galleria mellonella* as an alternative infection model for *Yersinia pseudotuberculosis*. Microbiology.

[11] Senior NJ (2011). *Galleria mellonella* as an infection model for *Campylobacter jejuni* virulence. J. Med. Microbiol..

[12] Alenizi D (2016). All *Yersinia enterocolitica* are pathogenic: Virulence of phylogroup 1 *Y.** enterocolitica* in a *Galleria mellonella* infection model. Microbiology.

[13] Barnoy S (2017). The *Galleria mellonella* larvae as an in vivo model for evaluation of *Shigella* virulence. Gut Microb..

[14] Lange A (2019). A *Galleria mellonella* oral administration model to study commensal-induced innate immune responses. J. Vis. Exp..

[15] Allonsius CN (2019). The microbiome of the invertebrate model host *Galleria mellonella* is dominated by *Enterococcus*. Anim. Microbiol..

[16] Gorodnichev RB (2021). Novel *Klebsiella pneumoniae* K23-specific bacteriophages from different families: Similarity of depolymerases and their therapeutic potential. Front. Microbiol..

[17] Insua JL (2013). Modeling *Klebsiella pneumoniae* pathogenesis by infection of the wax moth *Galleria mellonella*. Infect. Immun..

[18] Sugeçti S (2021). Pathophysiological effects of *Klebsiella pneumoniae* infection on *Galleria mellonella* as an invertebrate model organism. Arch. Microbiol..

[19] Thiry D (2019). New bacteriophages against emerging lineages ST23 and ST258 of *Klebsiella pneumoniae* and efficacy assessment in *Galleria mellonella* larvae. Viruses.

[20] Kilkenny C (2010). Improving bioscience research reporting: The arrive guidelines for reporting animal research. PLoS Biol..

[21] Glonti T (2022). In vitro techniques and measurements of phage characteristics that are important for phage therapy success. Viruses.

[22] Kropinski AM (2009). Enumeration of bacteriophages by double agar overlay plaque assay. Methods Mol. Biol..

[23] Dubourg G (2014). Culturomics and pyrosequencing evidence of the reduction in gut microbiota diversity in patients with broad-spectrum antibiotics. Int. J. Antimicrob. Agents.

[24] Blaser M (2011). Stop the killing of beneficial bacteria. Nature.

[25] Llor C (2014). Antimicrobial resistance: risk associated with antibiotic overuse and initiatives to reduce the problem. Ther. Adv. Drug Saf..

[26] Liu JY (2022). Decolonization of carbapenem-resistant *Klebsiella pneumoniae* from the intestinal microbiota of model mice by phages targeting two surface structures. Front. Microbiol..

