# Supplementary material for: Bacteriophage-mediated decolonization of Klebsiella pneumoniae in a novel Galleria mellonella gut colonization model with Enterobacteriaceae
Source: Sci Rep. 2024 Jan 3;14:318. doi: 10.1038/s41598-023-50823-9 (PMC10764950; doi:10.1038/s41598-023-50823-9)
Supplement: Supplementary file 1 — Supplementary Information. [file 41598_2023_50823_MOESM1_ESM.docx]

# Supplementary Data

**Table S1** Statistical analysis and significantly different groups of the study.

| **Target groups** | **Significance**  p<0.05 (*), p<0.01 (**), p<0.001 (***), p<0.0001(****) |
| --- | --- |
| **Figure 3a: Statistical analysis using Log-rank (Mantel-Cox) test** | |
| MH media vs 10^5^ CFU/larvae | * |
| MH media vs 10^6^ CFU/larvae | ** |
| 10^2^ CFU/larvae vs 10^5^ CFU/larvae | * |
| 10^2^ CFU/larvae vs 10^6^ CFU/larvae | ** |
| 10^4^ CFU/larvae vs 10^6^ CFU/larvae | * |
| **Figure 3b: Statistical analysis using the Kruskal-Wallis test** | |
| 10^2^ CFU/larvae (24h) vs 10^5^ CFU/larvae (24h) | **** |
| 10^2^ CFU/larvae (24h) vs 10^6^ CFU/larvae (24h) | ** |
| 10^2^ CFU/larvae (24h) vs 10^5^ CFU/larvae (48h) | * |
| 10^4^ CFU/larvae (24h) vs 10^5^ CFU/larvae (24h) | * |
| 10^3^ CFU/larvae (48h) vs 10^5^ CFU/larvae (24h) | * |
| **Figure 4: Statistical analysis using Log-rank (mantel-Cox) test** | |
| PBS vs 10^3^ CFU/larvae | * |
| PBS vs 10^4^ CFU/larvae | *** |
| PBS vs 10^5^ CFU/larvae | **** |
| PBS vs 10^6^ CFU/larvae | **** |
| 10^2^ CFU/larvae vs 10^4^ CFU/larvae | ** |
| 10^2^ CFU/larvae vs 10^5^ CFU/larvae | **** |
| 10^2^ CFU/larvae vs 10^6^ CFU/larvae | **** |
| 10^3^ CFU/larvae vs 10^5^ CFU/larvae | ** |
| 10^3^ CFU/larvae vs 10^6^ CFU/larvae | ** |
| 10^4^ CFU/larvae vs 10^5^ CFU/larvae | * |
| 10^4^ CFU/larvae vs 10^6^ CFU/larvae | * |
| **Figure 5a: Statistical analysis using Log-rank (mantel-Cox) test** | |
| MH media vs 10^5^ CFU/larvae ATCC 700603 | ** |
| **Figure 5b: Statistical analysis using Log-rank (mantel-Cox) test** | |
| MH media vs 10^5^ CFU/larvae ATCC 35218 | * |
| MH media vs 10^6^ CFU/larvae ATCC 35218 | *** |
| MH media vs 10^5^ CFU/larvae Ec 208873 | ** |
| MH media vs 10^6^ CFU/larvae Ec 208873 | * |
| MH media vs 10^5^ CFU/larvae Ec 280624 | * |
| **Figure 6a: Statistical analysis using Log-rank (mantel-Cox) test** | |
| MH media vs 10^6^ CFU/larvae ATCC 700603 | ** |
| MH media vs 10^6^ CFU/larvae Kp 419614 | ** |
| MH media vs 10^6^ CFU/larvae ATCC 35218 | * |
| MH media vs 10^6^ CFU/larvae Ec 208873 | **** |
| MH media vs 10^6^ CFU/larvae Ec 280624 | * |
| **Figure 7: Statistical analysis using the Kruskal-Wallis test** | |
| Untreated vs UZG4&13 (ATCC 700603) | ** |
| Untreated vs 0.1 mg/L meropenem (Kp 14520) | * |
| Untreated vs ciprofloxacin 32 mg/L (Kp 14520) | * |
| Untreated vs 32 mg/L ciprofloxacin (Kp 419614) | ** |
| Untreated vs UZG 4&13 (Kp 419614) | * |


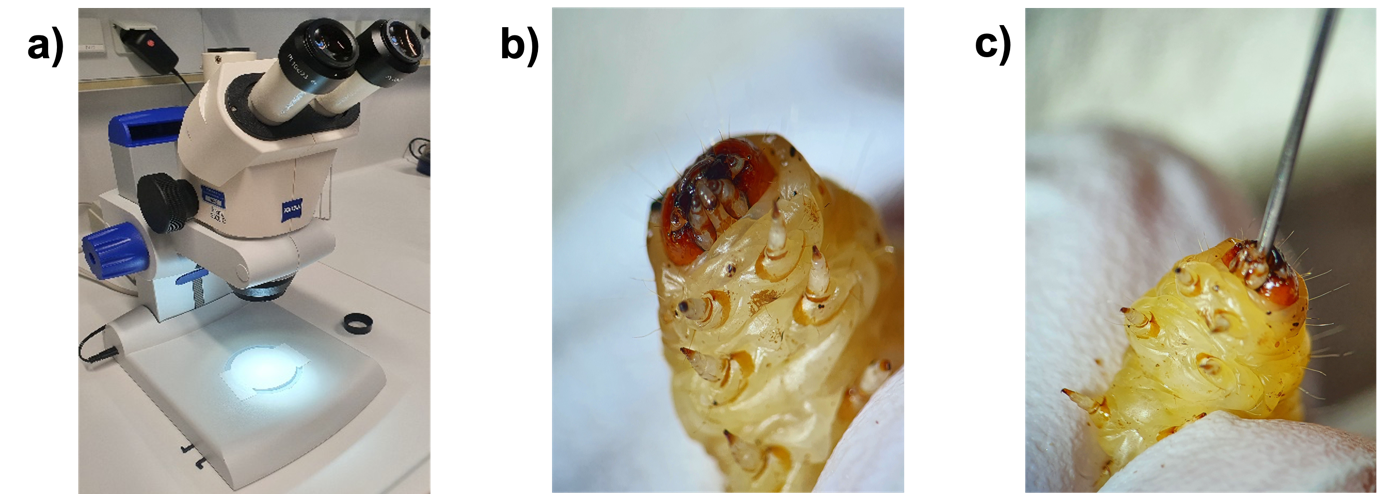


**Figure S1** (a) the Zeiss Stemi 2000-C zoom microscope. (b) the larvae's mouth under a microscope stereo-zoom microscope. (c) the larvae force-feeding under a microscope using a ALS blunt-end syringe.


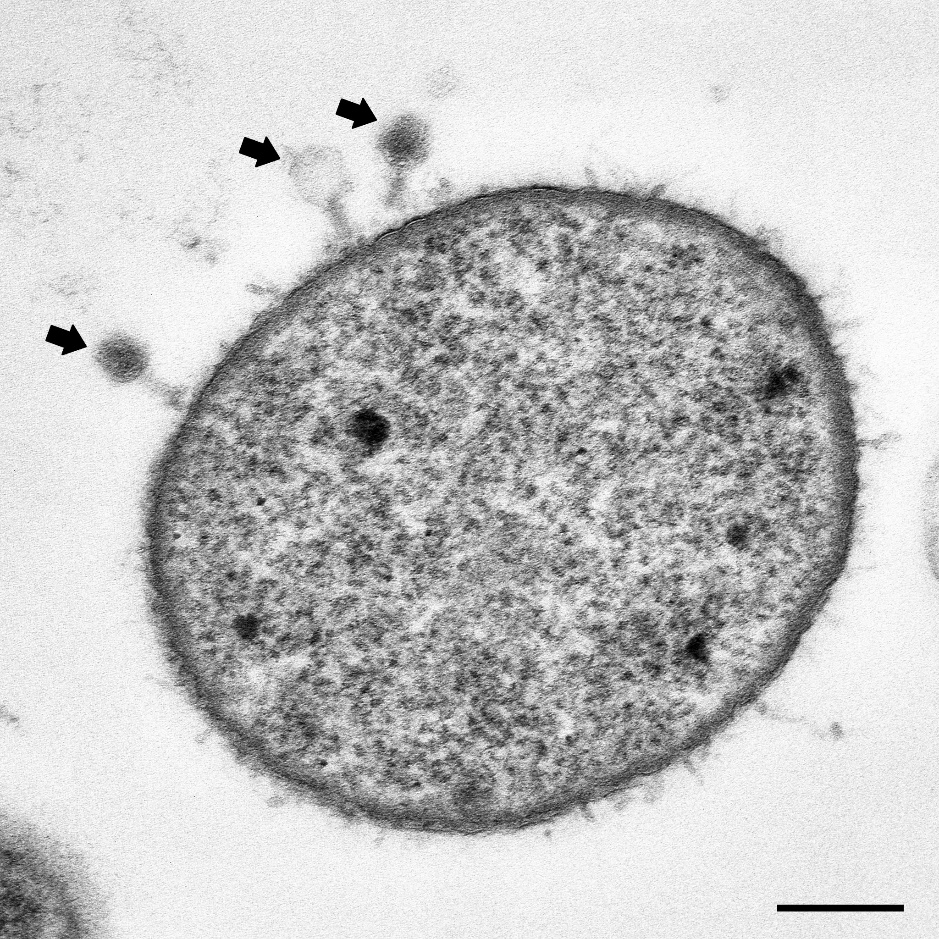


**Figure S2** Electron microscopic image of myoviridae bacteriophages UZG4 and UZG13 (arrows) attacking a *K. pneumoniae* ATCC 700603 bacterial cell. Magnification 85.000x, Scale bar: 200 nm


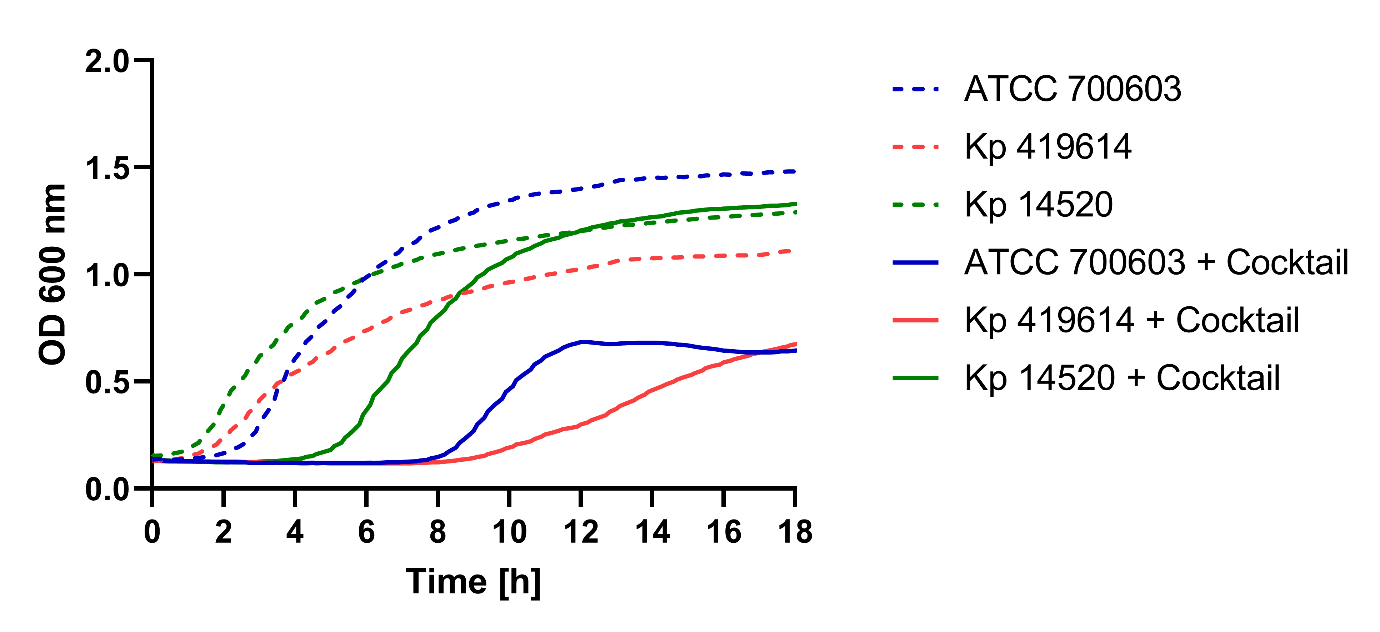


**Figure S3** The susceptibility of 10^7^ PFU UZG13 & UZG4 bacteriophage cocktail (solid lines) against the *K. pneumoniae* strains ATCC 700603, Kp 419614 and Kp 14520. The dotted lines represent the bacterial growth without phages (control).
